# Segregation of a Spontaneous *Klrd1* (CD94) Mutation in DBA/2 Mouse Substrains

**DOI:** 10.1534/g3.114.015164

**Published:** 2014-12-17

**Authors:** Dai-Lun Shin, Ashutosh K. Pandey, Jesse Dylan Ziebarth, Megan K. Mulligan, Robert W. Williams, Robert Geffers, Bastian Hatesuer, Klaus Schughart, Esther Wilk

**Affiliations:** * Department of Infection Genetics, Helmholtz Centre for Infection Research and University of Veterinary Medicine Hannover, Braunschweig, Germany; † Department of Genetics, Genomics and Informatics, Center for Integrative and Translational Genomics, University of Tennessee Health Science Center, Memphis, Tennessee; ‡ Research Group Genome Analytics, Helmholtz Centre for Infection Research, Braunschweig, Germany

**Keywords:** *Klrd1*, BXD, DBA/2

## Abstract

Current model DBA/2J (D2J) mice lack CD94 expression due to a deletion spanning the last coding exon of the *Klrd1* gene that occurred in the mid- to late 1980s. In contrast, DBA/2JRj (D2Rj) mice, crosses derived from DBA/2J before 1984, and C57BL/6J (B6) mice lack the deletion and have normal CD94 expression. For example, BXD lines (BXD1–32) generated in the 1970s by crossing B6 and D2J do not segregate for the exonic deletion and have high expression, whereas BXD lines 33 and greater were generated after 1990 are segregating for the deletion and have highly variable *Klrd1* expression. We performed quantitative trait locus analysis of *Klrd1* expression by using BXD lines with different generation times and found that the expression difference in *Klrd1* in the later BXD set is driven by a strong *cis*-acting expression quantitative trait locus. Although the *Klrd1*/CD94 locus is essential for mousepox resistance, the genetic variation among D2 substrains and the later set of BXD strains is not associated with susceptibility to the Influenza A virus PR8 strain. Substrains with nearly identical genetic backgrounds that are segregating functional variants such as the *Klrd1* deletion are useful genetic tools to investigate biological function.

DBA/2 (D2) is one of the oldest inbred strains of mice and has been used widely to study the genetic basis of many common diseases. This strain is also the paternal parent of the large family of C57BL/6J X DBA/2J (BXD) recombinant inbred strains (Peirce *et al.* 2004). In the early 1980s, D2 breeding stock from the Jackson Laboratory (DBA/2J; D2J) was transferred to the Zentralinstitut fuer Versuchstierzucht (Central Breeding Center for Laboratory Animals) in Hannover, Germany. In 1988, Janvier Breeding Centre acquired D2J stock from the Central Breeding Center for Laboratory Animals and bred them independently as DBA/2Rj (D2Rj) (Figure 1). In 2002, natural killer (NK) cells of D2J were discovered to lack expression of the CD94 (Cluster of Differentiation 94) gene (Vance *et al.* 2002). CD94 normally is expressed by NK cells and a subset of T cells, and this protein is encoded by the killer cell lectin-like receptor subfamily D, member 1 (*Klrd1*) gene on chromosome 6. CD94 forms heterodimers with NKG2 molecules displaying NK-cell receptors that bind to nonclassical major histocompatibility complex class I molecules. The loss of function in D2J mice is most likely caused by a 2.4-kb deletion of the last exon and 3′ end of *Klrd1* (Wilhelm *et al.* 2003). The deletion in *Klrd1* probably occurred between 1984 and 1989 at the Jackson Laboratory (see http://jaxmice.jax.org/jaxnotes/archive/495e.html and Figure 1).

**Figure 1:**
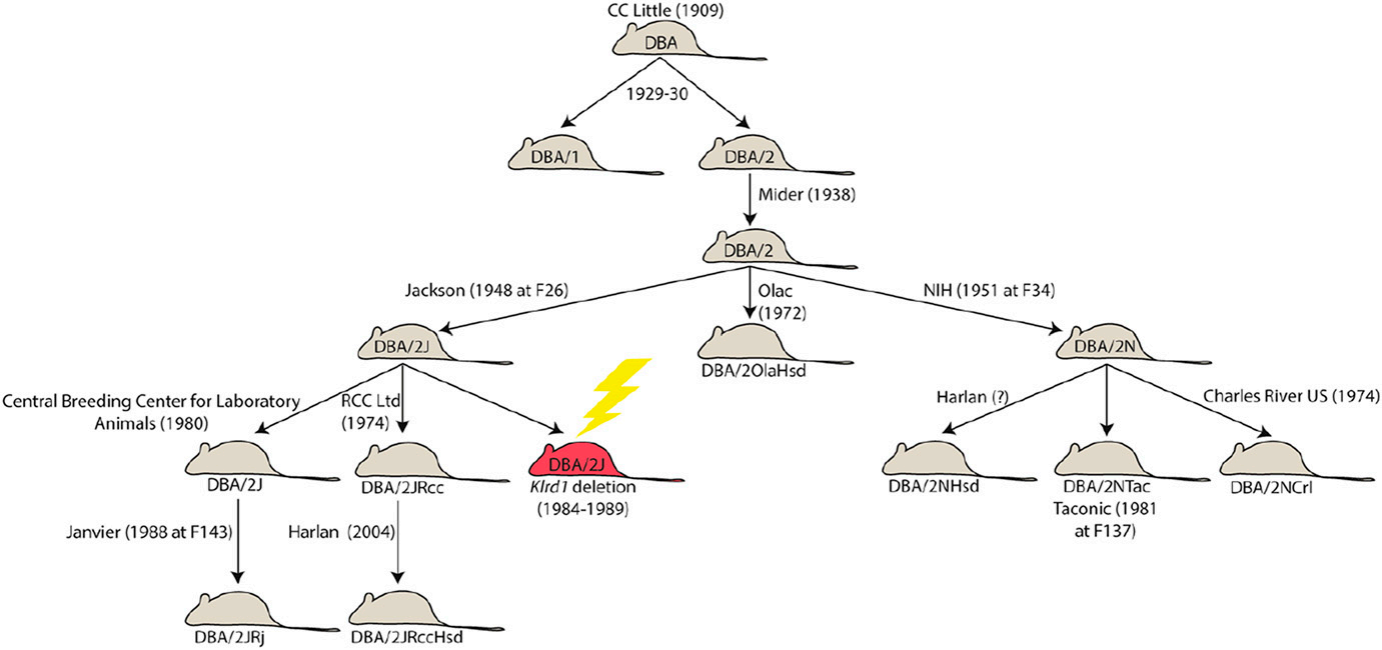
DBA/2 breeding history. D2 is the oldest inbred strain and originated as part of breeding efforts by C. C. Little around 1930. Since then, numerous D2 substrains were created by separation and breeding by different vendors, leading to genetic drift. These nearly identical lines create a valuable genetic resource for studying the downstream effects of spontaneous and naturally occurring mutations. In this case, a deletion (yellow lightning bolt) in the *Klrd1* gene occurred in the D2J substrain (red shading) between 1984 and 1989, leading to a loss of CD94 expression. Several substrains, including D2Rj, derived from the population at Jackson Laboratory before 1984 did not inherit the deletion. Information regarding substrain derivation dates was compiled from individual vendor Web sites.

We recently studied D2Rj stock from the Janvier Breeding Centre in France. In contrast to D2J from the Jackson Laboratory, D2Rj expresses CD94 protein (Figure 2A) on NK cells. We also found that the C57BL/6J (B6) strain has a greater number of NK cells expressing CD94 than D2Rj (Figure 2B).

**Figure 2:**
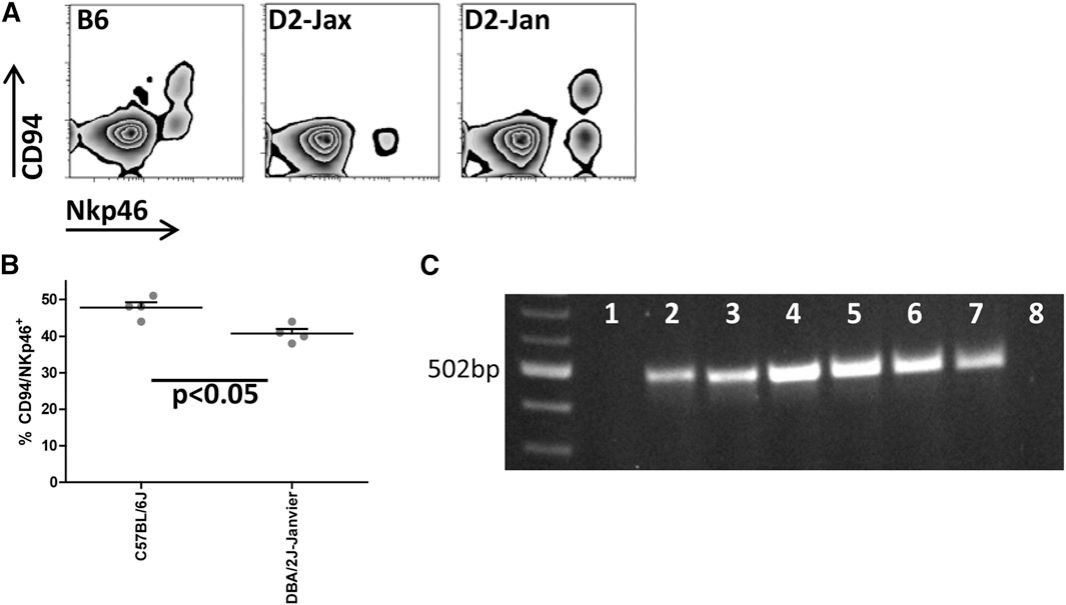
CD94 expression of D2 variants and B6. (A) CD94 expression was evaluated in peripheral blood by flow cytometry (Accuri C6, BD). Antibody staining was performed using NKp46-PerCP (eBioscience) as natural killer cell−specific marker and CD94-PE (BioLegend), respectively. Analysis was done with the software FlowJo. (B) Expression of CD94 determined by flow cytometry is significantly reduced in D2Rj mice compared with B6 (one representative experiment is shown, n = 4, females, 10-12 wk old; *P* < 0.05, Student’s *t*-test). (C) Amplification of genomic regions by polymerase chain reaction (PCR). DNA from D2J (lane 1), D2Rj (lane 2), D2Rj.2 (lane 3), B6 (lane 4), BXD9 (lane 5), BXD13 (lane 6), BXD31 (lane 7), and BXD98 (lane 8) was analyzed by PCR, using primers that hybridize to the presumed deleted region in D2J (fw-5′ tggccaggcaaagtgatacatacct; rev-5′acaatgcagtgctctggcctga).

To further investigate the basis of the difference in expression of CD94 in these D2 substrains, we analyzed high-throughput sequence data for D2J (~100X) and low coverage sequence data for D2Rj (5.5X). We confirmed the complete deletion of the last exon (exon 5) and 3′ UTR and a partial deletion of intron 4 of *Klrd1* in D2J (Figure 3B) using next-generation sequencing based on different structural variant detection approaches (Supporting Information, File S1). The discordant mate-pair approach detected a deletion on chr6 from 129,597,284 to 129,600,182 bp (mm10 assembly) and the read-depth approach detected a similarly sized deletion from 129,597,400 to 129,599,450 bp (mm10 assembly). However, we could not locate this deletion in D2Rj by using next-generation sequencing data. In addition, we performed polymerase chain reaction (PCR) analysis by using primers specific to exon 5—the key deleted region. No PCR product was detected in D2J, but we were able to amplify this exon in D2Rj (Figure 2C and Figure S1A). The sequence of the product showed 98% sequence identity with B6. The deletion in D2J starts within intron 5 (Figure 3A), consistent with previous findings (Wilhelm *et al.* 2003). In addition, both substrains contain a 191-bp deletion in the intronic region between exons 2 and 3. This second deletion was characterized by PCR (Figure 3, B and C and Figure S1B); D2J chr6:129,594,469-129,594,667, D2Rj chr6:129,594,271-129,594,389, mm10 assembly).

**Figure 3:**
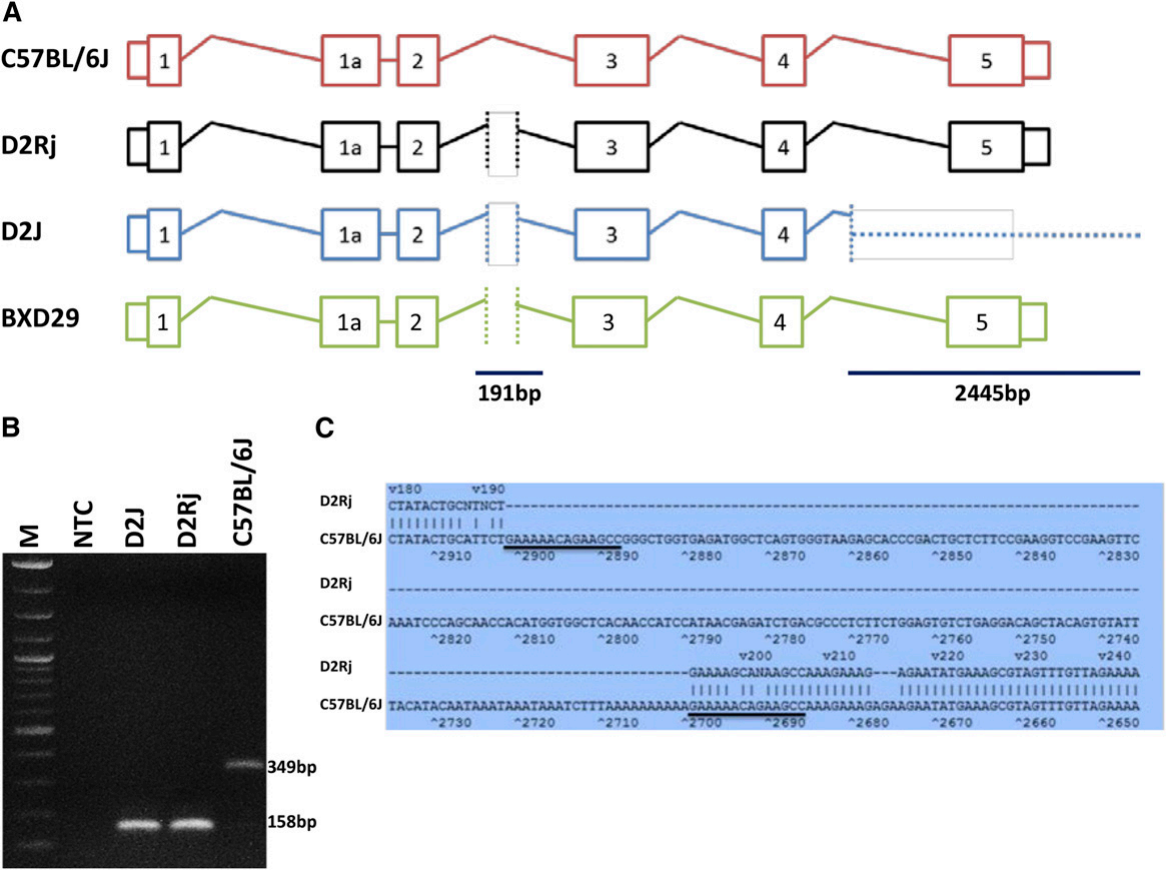
Characterization of the deletions in *Klrd1* gene in D2J and D2Rj. (A) Schematic representation of transcribed region at the *Klrd1* locus. Two deletions were observed in the *Klrd1* gene of D2J mice. The first deletion is located in intron 2, the second deletion starts in intron 4 and contains the entire exon 5. Both D2 strains carry the first deletion, whereas only D2J carries the downstream deletion that encodes a nonfunctional truncated gene product. In addition, sequence analysis of the BXD29 genome confirmed the structure of the “old” D2 allele that is carried by D2Rj mice: a deletion in intron 2 but not in exon 5. (B) Polymerase chain reaction (PCR) performed with genomic DNA from B6, D2J, and D2Rj mice detected the deletion in intron 2. DNA from both D2 strains generated a PCR product of 158 bp in length and B6 of 349 bp, respectively. Primers were as follows: fw-5′atacatgyttcctaacgagtgttc and rev-5′aaggtctattcttatagagatgtctatact. (C) In intron 2, a 191-bp deletion was observed in both D2 strains and a tandem repeat in B6 (underlined). Sequence alignment was performed with Martinez/Needleman-Wunsch method by MegAlign (DNASTAR, USA).

To explore the biological function and genetic regulation of the deletion in *Klrd1*, we levered the BXD family of strains. The first set of BXDs (BXD1−BXD32) was generated from intercrosses of B6 and D2J in the early 1970s—before the occurrence of the deletion (Taylor *et al.* 1973, 1975; Womack *et al.* 1975). Many more BXD strains (BXD33−BXD102) were initiated starting in the 1990s using D2J stock (Pandey and Williams 2014) that was already homozygous for the spontaneous *Klrd1* deletion (see http://www.genenetwork.org/mouseCross.html for more detailed information). As a result, the first set of BXDs does not segregate for the deletion whereas the second set does. As a result, members of the second set that inherited the D2 haplotype at *Klrd1* have very low expression (Figure 4A), and it is only in this latter set that *Klrd1* maps as a strong *cis*-acting expression quantitative trait locus (Figure 4B). The *cis*-acting expression quantitative trait locus has an effect size of 0.7 log_2_ expression units per allele, roughly equivalent to a 2.6-fold reduction of *Klrd1* mRNA. To confirm the segregation of the ancestral and *de novo* large deletion among BXD sets we sequenced BXD29—a member of the first epoch of BXDs that should have the original D2 haplotype at *Klrd1*. We confirmed that the last exon and the 3′ untranslated region were indeed intact. Similar to both D2J and D2Rj substrains, the 191-bp deletion in the intron is also detected in BXD29 (Figure 3A and Figure S1B).

**Figure 4:**
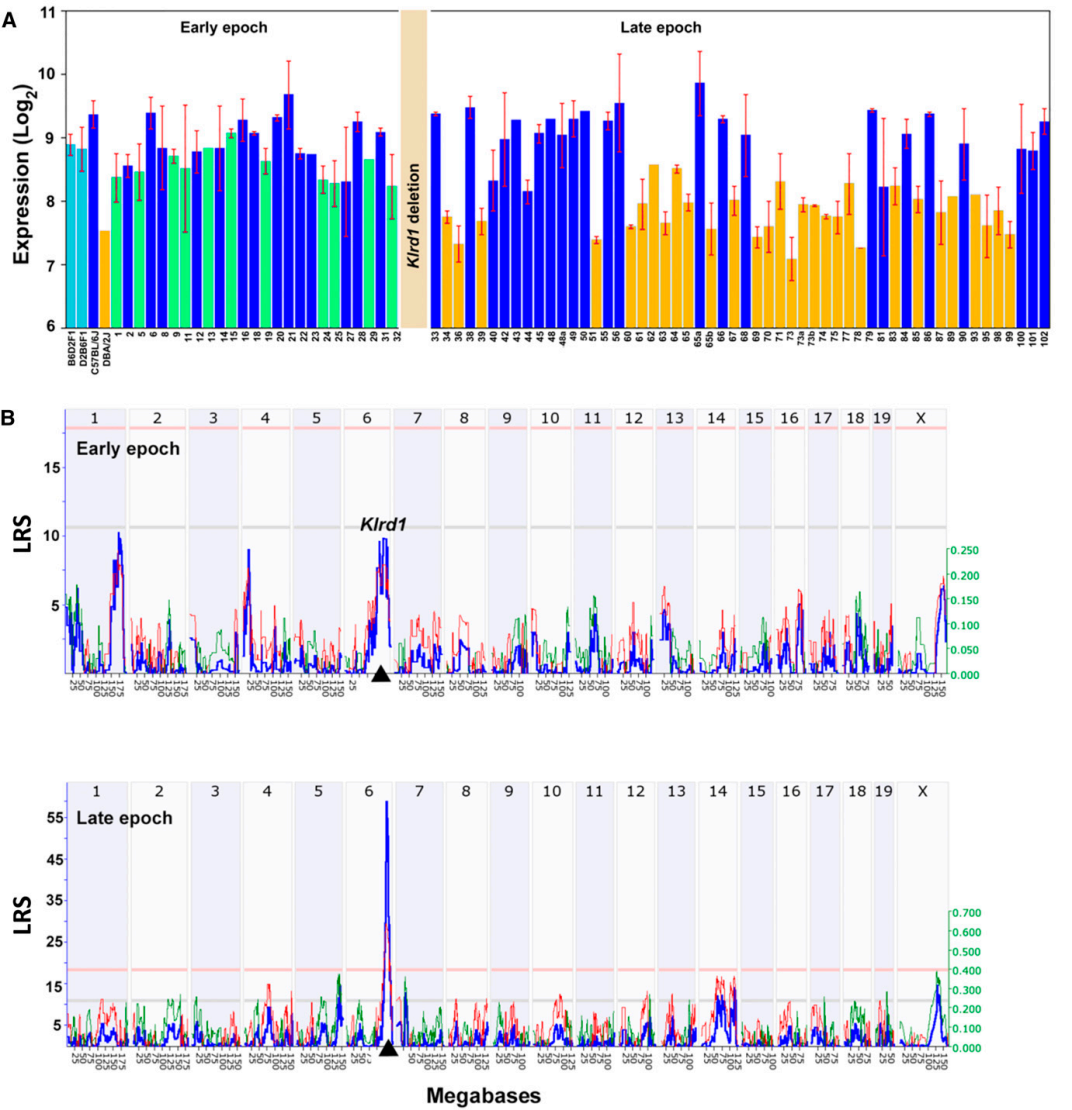
Variation in *Klrd1* expression in the spleen across BXDs. (A) *Klrd1* expression differences between early and late epochs of BXDs. Early epoch consists of BXD strains (1 through 32) that were generated using D2 strain before the *Klrd*1 deletion. Late epoch consists of BXD strains (33 through 102) that have been generated using D2 strain with deleted *Klrd1* locus. BXDs with the D2 haplotype at *Klrd1* have been shown in green and orange colors in early and late epochs respectively. BXDs with *B6* haplotype at *Klrd1* in both epochs have been shown in blue color. (B) *cis*-acting expression quantitative trait locus mapping of *Klrd1* expression using early BXD epoch (top) and the late BXD epoch (bottom). Expression variation in *Klrd1* maps significantly to the location of the gene itself (black triangle on the x-axis) in late BXD epoch (likelihood ratio statistic, LRS ~60). The numbers along the top of each plot represent chromosomes. The y-axis and the bold blue function provides the likelihood ratio statistic (LRS = 4.6 × LOD (log of the odds ratio)). The two horizontal lines across these plots mark genome significance thresholds at *P* < 0.05 (genome-wide significant, red line) and suggestive threshold (*P* < 0.63, gray line). The thin red and green functions summarize the average additive effects of D and B alleles among all BXD strains at particular markers. If BXD strains with a D allele have higher values than those with a B allele at a particular marker then the line is colored green. In contrast, if strains with the B allele have greater mean values, the line is colored red. This additive effect size is measure in log_2_ units per allele. In other words, an additive effect of 0.5 signifies a two fold difference in expression level between strains with BB and DD genotypes at a marker (log 2 raised to the power of 2×0.5).

In summary, D2 substrains differ greatly in *Klrd1* gene structure and expression (Figure 1). The differences between substrains from JAX (D2J) and Janvier (D2Rj) and the difference in CD94 expression represent a valuable resource for functional studies of CD94. Whereas CD94 is essential for NK cell−mediated resistance to mousepox (Fang *et al.* 2011) we did not detect a significant QTL at the *Klrd1* locus after influenza A infection with the mouse-adapted H1N1 virus PR8 (A/PuertoRico/8/34) (Nedelko *et al.* 2012). Similarly, no significant phenotypic differences were found between D2J and D2Rj after infection with PR8.

## Supplementary Material

Supporting Information
