# Supplementary material for: Segregation of a Spontaneous Klrd1 (CD94) Mutation in DBA/2 Mouse Substrains
Source: G3 (Bethesda). 2014 Dec 17;5(2):235–9. doi: 10.1534/g3.114.015164 (PMC4321031; doi:10.1534/g3.114.015164)
Supplement: Supporting Information [file supp_5_2_235__index.html]

Segregation of a Spontaneous Klrd1 (CD94) Mutation in DBA/2 Mouse Substrains — Supporting Information 

# Segregation of a Spontaneous *Klrd1* (CD94) Mutation in DBA/2 Mouse Substrains

## Supporting Information for Shin *et al.*, 2015

**Files in this Data Supplement:**

- Supporting Information - File S1 and Figure S1 (PDF, 319 KB)
- File S1 - Material and Methods (PDF, 175 KB)
- Figure S1 - *Klrd1* alleles in BXD mouse strains. (PDF, 243 KB)
