# Supplementary material for: Segregation of a Spontaneous Klrd1 (CD94) Mutation in DBA/2 Mouse Substrains
Source: G3 (Bethesda). 2014 Dec 17;5(2):235–9. doi: 10.1534/g3.114.015164 (PMC4321031; doi:10.1534/g3.114.015164)
Supplement: Supporting Information [file supp_g3.114.015164_FileS1.pdf]

File S1

Material and Methods

Sequence Analysis of DBA/2J (D2J)

Data collection for DBA/2J (D2J). We downloaded the paired-end sequencing data for D2J mouse strain from the European Nucleotide Archive (ENA), accession number ERP000044 (KEANE *et al.* 2011) and ERP000927. ERP000044 sequencing data consists of 9 Illumina paired-end libraries sequenced on Illumina GAII platform. The read lengths for these libraries varies between 54-76 nt. ERP000927 sequencing data consists of 2 Illumina paired-end libraries sequenced on Illumina HiSeq with read length of 100 nt. Additionally, we also downloaded Illumina paired-end sequencing data from SRA, accession number SRP001135. This data consists of 3 libraries sequenced on Illumina GAII with read length of 100 nt.

Library preparation and sequencing of BXD29. 1ug of the genomic DNA was used to prepare libraries for sequencing using the AB Mate Pair library Kit for 5500 Genetic Analysis Systems. The libraries were amplified 10 cycles and then quantified on an Agilent high sensitivity DNA chip and by real time PCR using the Life Technologies Library Quantification Kit and a Roche LC480 system. These libraries were used to prepare beads using the Life Technologies EZ bead system at (MRC) Molecular Resource Centre at University of Tennessee Health Science Center. Beads prepared were then sequenced at MRC on a 5500XL sequencer using mate pair chemistry.

Read alignment and post-alignment processing. For Illumina reads, sequencing reads from each lane were trimmed off for the low quality bases and aligned to the C57BL/6J reference genome (mm10) using Burrows Wheeler Aligner(LI and DURBIN 2010) (version 6.1) and the parameters "-q 15". Quality scores were recalibrated at the lane level using Genome Analysis Toolkit (MCKENNA *et al.* 2010) (GATK version 2.7) 'TableRecalibration'. All lanes from the same library were then merged together into a single BAM file using Picard tools (version 1.8, http://picard.sourceforge.net/). PCR duplicates were flagged at the library level using Picard 'MarkDuplicates'. BAM files representing each library were merged together to create a single BAM file containing all the DBA/2J sequences. Finally, GATK 'IndelRealigner' was used to realign reads near indels from the Mouse Genome Project (KEANE *et al.* 2011) as well as potential Indels predicted by GATK.

For SOLiD reads, 5500xl SOLiD mate-paired reads were aligned using Life Technologies' proprietary Lifescope (version 2.1, http://www.lifetechnologies.com/us/en/home/technical-resources/software-downloads/lifescope-genomic-analysis-software.html) software. Reads with mapping quality of less than 10 were discarded. Aligned reads from different lanes were merged into a single BAM file and duplicate reads were removed using the Picard tools.

Structural variant discovery and annotation. For Illumina reads,structural variants were identified using three different methods including discordant mate-pair analysis (BreakDancer (CHEN *et al.* 2009)), read-depth (CND (SIMPSON *et al.* 2010)) and split-read analysis (Pindel (YE *et al.* 2009)). BreakdancerMax was run using the following parameters '-c 3 -m 10000000 -q 25 -r 3 -h –f'. CND was run using the default settings. Pindel were run using the following settings '-e 3 -f 1000 -sb -ss 1 –G'. In-house python scripts were used to annotate structural variants and the affected genes.

For SOLiD reads, Lifescope large indel variation detection module was used to call for structural variants. This module identifies large indels from 100bp to 100Kb with high confidence. CND tool was run using the default settings. In-house python scripts were used to annotate structural variants and the affected genes.

Sequence Analysis of DBA/2JRj (D2Rj)

Illumina Sequencing-HZI, Braunschweig. Library preparation for whole genome sequencing was done using TruSeq DNA Sample Prep Kit (llumina Inc., San Diego, CA, USA, Cat: FC-121-2001) following manufacturer's instruction. Briefly, 1 µg of genomic DNA was fragmented using a Covaris S2 (Covaris, MA, USA) to approximately 400 bp. The double stranded DNA fragments comprised of 3' or 5' overhangs were converted to blunt ends with an 'A' base using End Repair Mix 2 for adaptor ligation. The resulting fragments were purified using AMPure XP beads followed by adenlytion of 3`end. Adapters were then ligated to the adenylated fragments. The resulting ligation product was again purified from Agarose gel. Enrichment of DNA fragment was done for 10 PCR cycles. The final library was purified using AMPure XP beads. Quality control of the amplified libraries was validated using Agilent Bioanalyzer HS Chip (Agilent Technologies) following the manufacturer's instruction. Cluster generation was performed with cBot (Illumina) using TruSeq PE Cluster Kit v3–cBot-HS (Illumina). Sequencing was done on the HiSeq 2500 (Illumina) using TruSeq Rapid SBS Kit v3 - HS (Illumina) for 100 cycles in both directions in high output mode. Image analysis and base calling were performed using the Illumina pipeline v 1.8.resulting in approximately 143 Mio reads and 14,5 Gbases of sequence information. The average coverage of the murine genome (mm9 , reference genome: 2,6 Gbases) was 5.5 X. Alignment of short reads was performed with the Genomic Workbench 5.5 (Qiagen, Hilden, Germany).99.25% of total reads could be mapped to the murine reference genome (mm9). The results can be found in European Nucleotide Archive (EMBL-EBI) under accession number ERS538351.

Array expression data and eQTL mapping

For differential cis-eQTL and expression analyses between early and late epochs (Figure 4), we used an Affymetrix dataset consisting of spleen gene expression data for 81 genetically diverse BXD strains (GeneNetwork accession GN283). We performed robust multichip analysis (RMA) preprocessing and rescaled values to $\log_2$ and stabilized the variance across samples. Expression levels below 6 are close to background noise levels. We used GeneNetwork.org, a web-based tool to map the expression variation in *Klrd1* gene.

CHEN, K., J. W. WALLIS, M. D. MCLELLAN, D. E. LARSON, J. M. KALICKI *et al.*, 2009 BreakDancer: an algorithm for high-resolution mapping of genomic structural variation. Nat Methods **6:** 677-681.

KEANE, T. M., L. GOODSTADT, P. DANECEK, M. A. WHITE, K. WONG *et al.*, 2011 Mouse genomic variation and its effect on phenotypes and gene regulation. Nature **477:** 289-294.

LI, H., and R. DURBIN, 2010 Fast and accurate long-read alignment with Burrows-Wheeler transform. Bioinformatics **26:** 589-595.

MCKENNA, A., M. HANNA, E. BANKS, A. SIVACHENKO, K. CIBULSKIS *et al.*, 2010 The Genome Analysis Toolkit: a MapReduce framework for analyzing next-generation DNA sequencing data. Genome Res **20:** 1297-1303.

SIMPSON, J. T., R. E. MCINTYRE, D. J. ADAMS and R. DURBIN, 2010 Copy number variant detection in inbred strains from short read sequence data. Bioinformatics **26:** 565-567.

YE, K., M. H. SCHULZ, Q. LONG, R. APWEILER and Z. NING, 2009 Pindel: a pattern growth approach to detect break points of large deletions and medium sized insertions from paired-end short reads. Bioinformatics **25:** 2865-2871.
