# Supplementary material for: Segregation of a Spontaneous Klrd1 (CD94) Mutation in DBA/2 Mouse Substrains
Source: G3 (Bethesda). 2014 Dec 17;5(2):235–9. doi: 10.1534/g3.114.015164 (PMC4321031; doi:10.1534/g3.114.015164)
Supplement: Supporting Information [file supp_g3.114.015164_FigureS1.pdf]

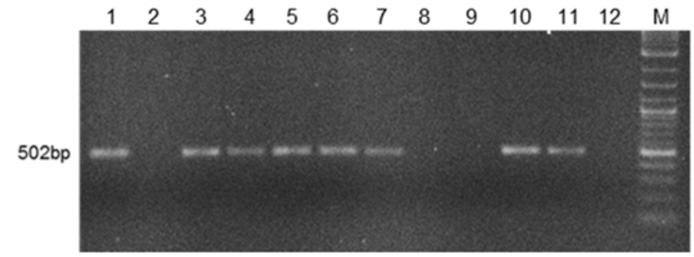

(A)

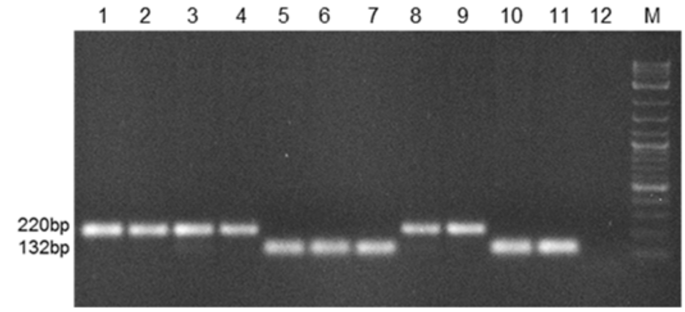

(B)

Figure S1 *Klrd1* alleles in BXD mouse strains. Lane 1: D2Rj; 2: D2J; 3: BXD9; 4: BXD13; 5: BXD31; 6: BXD45; 7: BXD55; 8: BXD65; 9: BXD98; 10: B6; 11: Serpine1-ko mice with C57BL/6J background; 12: NTC; M: DNA ladder.

A. PCR within intron 5 of *Klrd1*. BXD 9 and 13 carried the D2Rj allele displaying the old D2 genotype. Late BXD lines 65 and 98 with the D2J allele contained the deletion. BXD 31, 43 and 55 had the C57BL/6J allele. Primers used: 5’-tggccaggcaaagtgatacatacct-3’ and 5’-acaatgcagtgctctggcctga-3’.

B. The deleted region in intron 2 of *Klrd1* was analyzed by PCR distinguishing the DBA/2J (220bp) or C57BL/6J (132bp) allele. BXD 31, 43 and 55 carried the C57BL/6J allele whereas BXD 9, 13, 65 and 98 had the DBA/2J allele. Primers were as follow: i2-fwd: 5’-aaagtctcccataaaattgtcatcat-3’, i2-rev1: 5’-aaggtctattcttatagagatgtctatact, i2-rev2: 5’-catgtggttgctgggatttg.
